# Extreme prematurity and sepsis strongly influence frequencies and functional characteristics of circulating γδ T and natural killer cells

**DOI:** 10.1002/cti2.1294

**Published:** 2021-06-10

**Authors:** Khaleda Rahman Qazi, Georg B Jensen, Marieke van der Heiden, Sophia Björkander, Giovanna Marchini, Maria C Jenmalm, Thomas Abrahamsson, Eva Sverremark‐Ekström

**Affiliations:** ^1^ Department of Molecular Biosciences The Wenner‐Gren Institute Stockholm University Stockholm Sweden; ^2^ Department of Biomedical and Clinical Sciences Linköping University Linköping Sweden; ^3^ Department of Paediatrics Linköping University Linköping Sweden; ^4^ Department of Women's and Children's Health Karolinska Institutet Stockholm Sweden

**Keywords:** extreme preterm, gestational age, natural killer cells, neonatal immunity, sepsis, unconventional T cells

## Abstract

**Objectives:**

Extremely low gestational age neonates with extremely low birthweight (ELGAN/ELBW) are highly susceptible to infection. This is linked to their relatively immature immune system which is not yet fully compatible with an extra‐uterine environment. Here, we performed a longitudinal characterisation of unconventional T and natural killer (NK) cells in ELGAN/ELBW during their first months of life.

**Methods:**

Peripheral blood mononuclear cells were collected from 97 ELGAN/ELBW at 14 and 28 days of life and at a time point corresponding to postmenstrual week 36 + 0. γδ T‐cell, NKT‐cell, mucosa‐associated invariant T‐cell and NK cell frequencies and characteristics were analysed by flow cytometry. As control, cells from 14‐day‐old full‐term (FT) infants were included.

**Results:**

Extreme prematurity had significant bearing on γδ T‐cell and NK cell frequencies and characteristics. ELGAN/ELBW had significantly higher proportions of γδ T cells that were skewed towards effector and effector memory phenotypes, characteristics that were maintained throughout the study period. Expression of the gut homing receptor CCR9 was also more common in γδ T cells from ELGAN/ELBW. Conversely, NK cell frequencies were markedly lower and skewed towards a cytotoxic phenotype in the ELGAN/ELBW group at 14 days of age. Culture‐proven sepsis with an onset during the first 14 days after birth further manifested these differences in the γδ T‐ and NK cell populations at 14 days of age.

**Conclusion:**

Prematurity strongly influences the levels of γδ T and NK cells, in particular in cases where sepsis debuts during the first 2 weeks of life.

## Graphical abstract

Extreme prematurity impacts frequencies and characteristics of γδ T and NK cells, in particular in cases where sepsis debuts during the first 2 weeks of life. Probiotic supplementation with *Lactobacillus* *reuteri* did not influence these parameters.

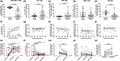

## Introduction

Preterm birth is a strong risk factor for neonatal disease, long‐term complications and death. Although the care of preterm infants has improved dramatically during the last decades, still more than 20% of extremely low gestational age neonates (< week 28) with extremely low birthweight (< 1000 g) (ELGAN/ELBW) die within the first year of life.^1^

While full‐term neonates have a relatively well‐developed immune system,^2^ preterm neonates have an impaired ability to combat pathogens, with invasive infection and sepsis as leading causes of morbidity and mortality.^3^ This is clearly linked to features of their immune system that is still developing: while it is probably appropriate for their respective developmental stage, it is not fully compatible with an extra‐uterine life.

Innate lymphocytes such as natural killer (NK), NKT, γδ T and mucosa‐associated invariant T (MAIT) cells all develop and mature during foetal life, but at different time points. Although they are considered to be involved in innate immune responses, their functions are still attenuated and/or different in newborns compared with adults.^4^, ^5^, ^6^, ^7^, ^8^, ^9^, ^10^ During foetal life, NK cells are highly regulated and hypo‐responsive but are present at high frequencies in cord blood.^11^, ^12^ γδ T cells are relatively abundant in early life and have been suggested to be a particularly important constituent of the CD3^+^ T‐cell pool during this period when the conventional αβ T cells are not fully functional.^5^, ^13^, ^14^ Human NKT cells can differentiate and functionally mature before birth^15^ but are usually found in low frequencies in the circulation.^6^ MAIT cells are a highly conserved population of T lymphocytes and represent a very small fraction of cord blood T cells, but can comprise up to 10% of the entire T‐cell population in adults.^7^, ^8^

In extremely premature neonates, the conventional T‐cell compartment is compromised at birth, and they may therefore rely heavily on their unconventional T cells and NK cells in order to successfully clear infections in the neonatal period.^13^, ^14^, ^15^, ^16^ Still, prematurity impacts most immune compartments, including NK cell and NKT‐cell frequencies,^12^, ^17^, ^18^ as well the functional abilities of γδ T cells.^13^

However, comprehensive longitudinal studies on the composition of unconventional T and NK cells in ELGAN/ELBW during their first weeks of life and their role in clinical outcomes are lacking. Therefore, we performed a longitudinal characterisation of these peripheral innate lymphocytes in ELGAN/ELBW during their first months of life. The relative frequencies and functional phenotypes of these cell types were correlated with gestational age at birth, pregnancy complications such as preeclampsia and chorioamnionitis, and neonatal sepsis, as well as with postnatal probiotic supplementation.

## Results

### Unconventional T‐cell and NK cell compositions are altered in ELGAN/ELBW neonates

**Figure 1:**
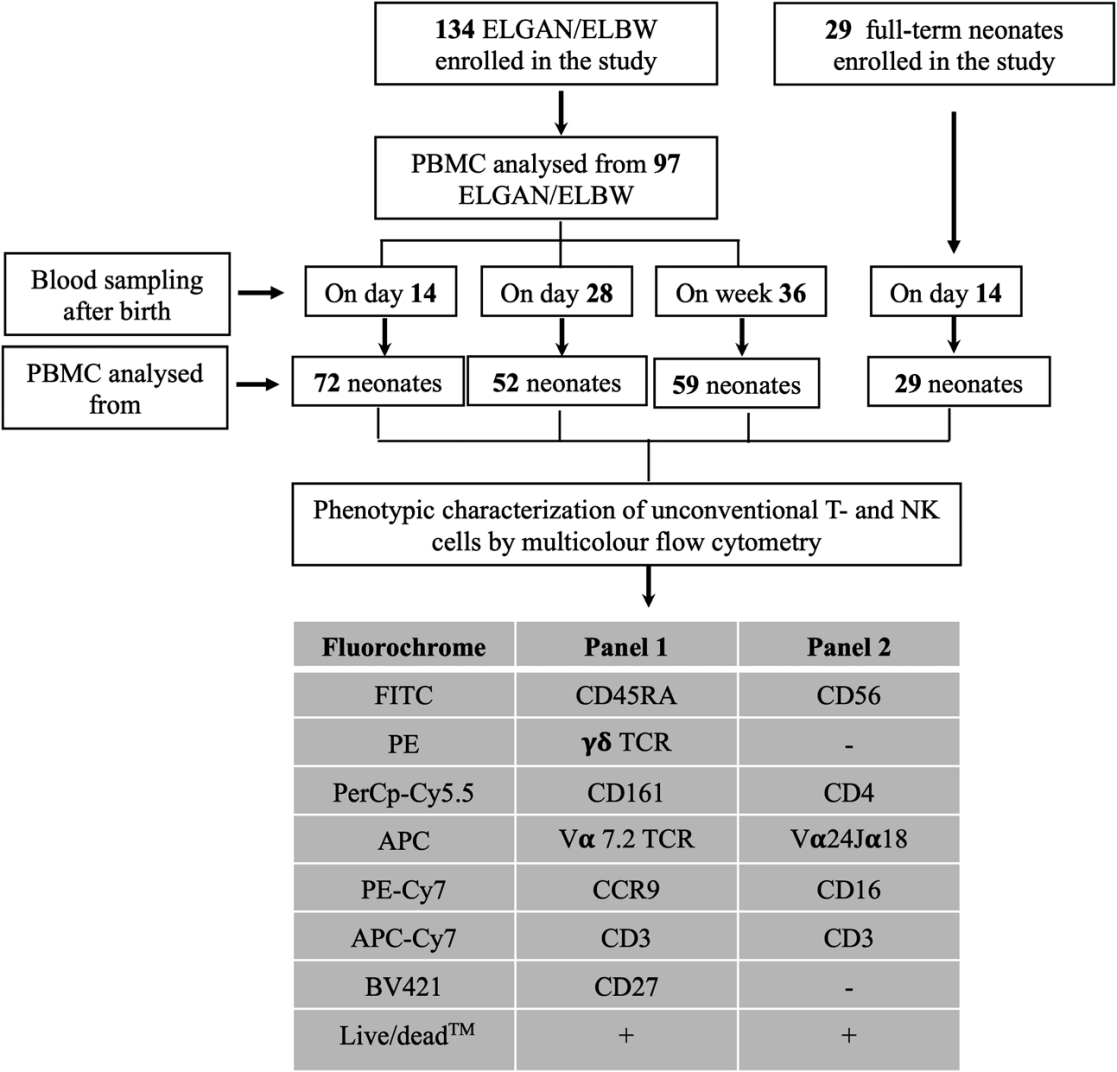
Flow chart of the number of subjects recruited, time points for blood sampling, number of PBMC samples analysed by flow cytometry and the antibody panels used for the experiments to characterise NK, NKT γδ T and MAIT cells. The final number of PBMC samples analysed for each parameter were dependent on cell numbers and viability, as indicated in each of the subsequent figures.

**Figure 2:**
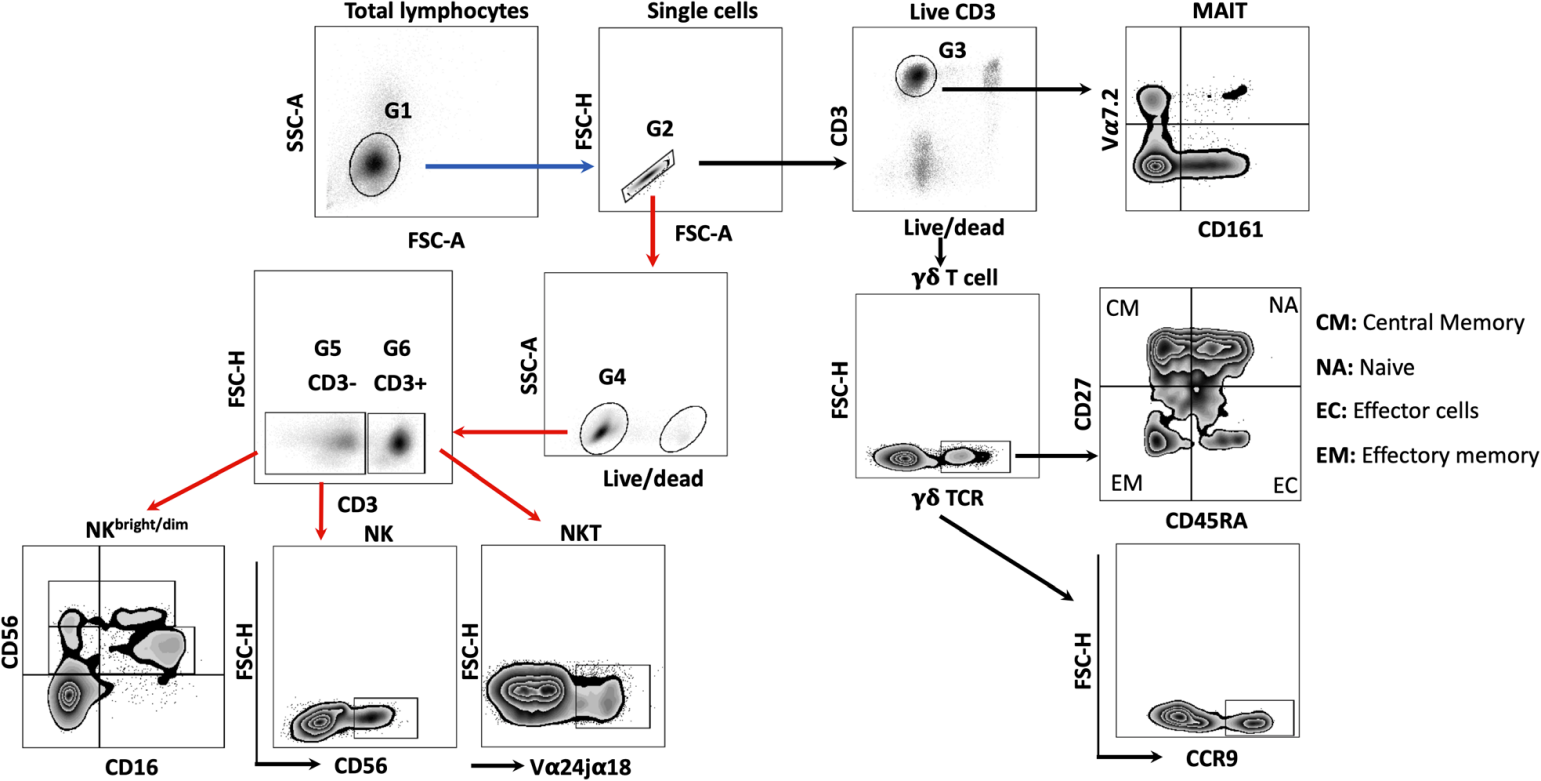
Schematic presentation of the gating strategies used for the identification and characterisations of the NK cells and unconventional T cells and subsets. NK/NKT cells and γδ T‐/MAIT cells were analysed in two different panels as indicated in the flow chart with red and black arrows, respectively. Lymphocytes (G1) were gated based on their physical properties as side scatter (SSC) and forward scatter (FSC) followed by gating on single cells (G2). From G2 gate, cells were either divided as live CD3^+^ cells (G3) (black arrow) or as live lymphocytes (G4) (red arrow), which were further gated on the basis of the cell population‐specific markers as pan γδ TCR^+^ cells (γδ T cells) and CD161^+^Vα7.2^+^ cells (MAIT cells) (black arrows) or as CD3^+^ Vα24jα18^+^ cells (NKT cells) and CD3^‐^CD56^+^ cells (total NK cells)/CD56^+^CD16^+^(NK^bright/dim^) (red arrows), respectively. γδ T cells were further analysed for either the subpopulations (CM, NA, EC and EM) or the expression of the molecule as CCR9.

**Figure 3:**
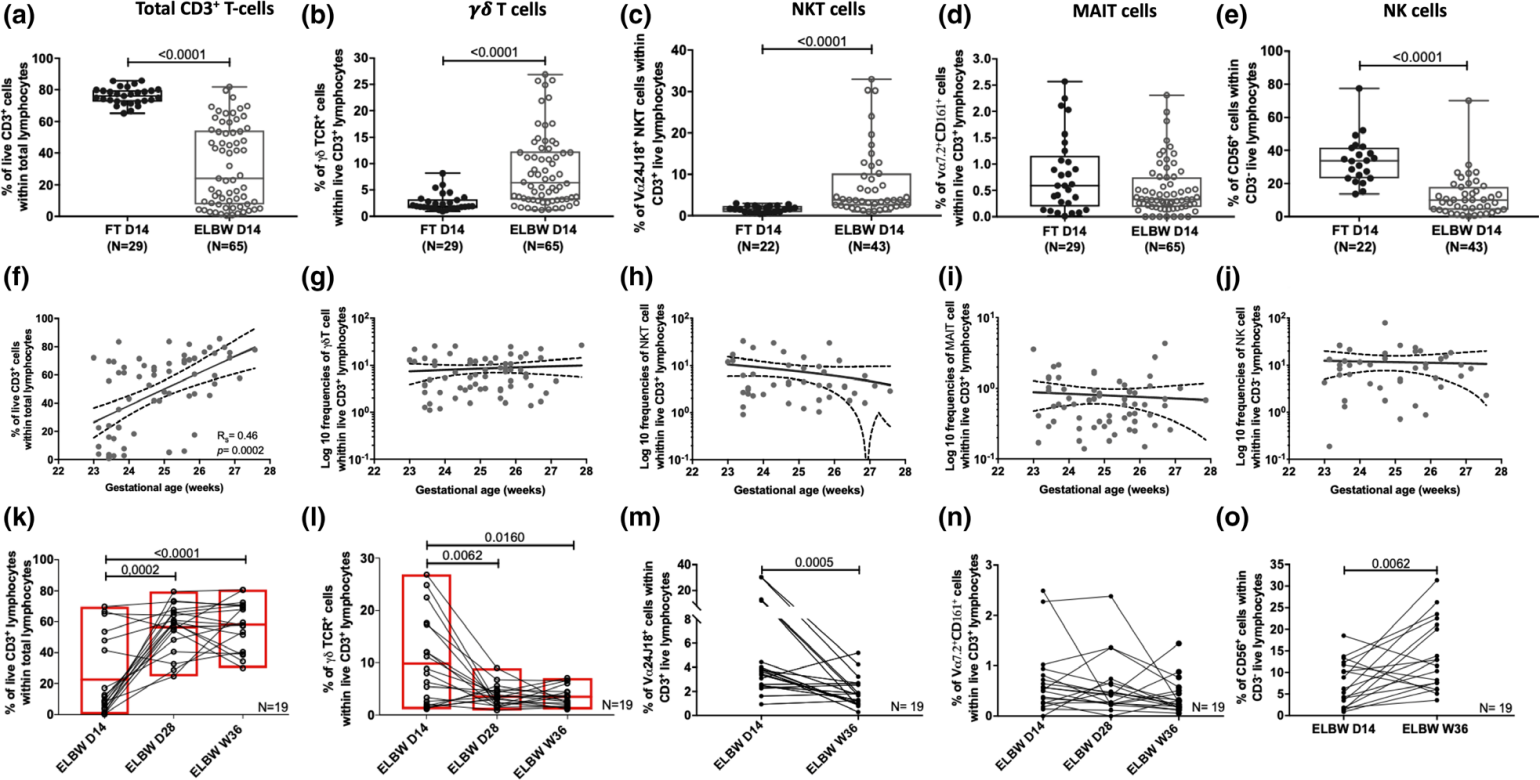
Peripheral γδ T‐, NKT‐cell and NK cell frequencies are altered by extreme prematurity. Frequencies of **(a)** CD3^+^, **(b)** γδ TCR^+^, **(c)** NKT, **(d)** MAIT, and **(e)** NK cells among live lymphocytes in 14‐day‐old ELGAN/ELBW and FT infants are shown. The correlations between gestational age at birth and the percentages of **(f)** CD3^+^, **(g)** γδ T, **(h)** NKT, **(i)** MAIT and **(j)** NK cells at 14 days of life are shown for the ELGAN/ELBW. The postnatal development of these populations is shown as proportions of **(k)** CD3^+^, **(l)** γδ T, **(m)** NKT, **(n)** MAIT and **(o)** NK cells in longitudinal paired samples from ELGAN/ELBW at D14, D28 and W36 **(k, l, n)** or D14 and W36 **(m, o),** respectively. The Mann–Whitney *U‐*test for unpaired samples and the Wilcoxon signed‐rank test for paired samples were used for group comparisons. Box and whisker plots show median as the central line and error bars represent minimum to maximum values. The line with error bars shows mean and error where the Spearman correlation test was used to analyse correlation between gestational age and the proportions of the different cell types analysed. Symbols and lines were overlaid on floating bars plotted with line at mean **(k, l)**.

The study design and antibody panels as well as gating strategies used are shown in Figures 1, 2, respectively. The total CD3^+^ T‐cell frequency was significantly lower in the ELGAN/ELBW group than in the FT neonates at 14 days of age (Figure 3a). This was likely to reflect a low percentage of conventional T cells, as both γδ T‐ and NKT‐cell frequencies constituted a proportionally large part of the CD3^+^ T‐cell pool in the ELGAN/ELBW compared with FT neonates (Figure 3b and c). The percentage of peripheral MAIT cells was very low in general, but were largely unaffected by prematurity (Figure 3d). In contrast, the proportion of NK cells was markedly lower in ELGAN/ELBW than in FT neonates (Figure 3e).

While the proportion of total CD3^+^ T cells clearly correlated with gestational age in 14‐day‐old ELGAN/ELBW (Figure 3f), this was not observed for any of the unconventional lymphocytes investigated (Figure 3g–j). Further, longitudinal analysis of paired samples within the ELGAN/ELBW group revealed that although the proportions of CD3^+^ T cells already increased significantly at 28 days of age (Figure 3k), the extremely premature neonates remained relatively T cell‐compromised also at week 36 (Figure 3k, Supplementary table 1). For γδ T and NKT cells, the proportions decreased with age (Figure 3l and m), and at week 36, they were proportional to percentages seen in FT neonates (Supplementary table 1). NK cell frequencies had increased significantly in ELGAN/ELBW infants at week 36, but were still low compared to the FT control group (Figure 3o, Supplementary table 2).

ELGAN/ELBW are occasionally treated with corticosteroids (betamethasone) to dampen inflammatory complications and increase their lung function in order to get them off the respirator. To rule out a significant impact of the corticosteroid treatment, we analysed the frequencies of viable CD3^+^, NK and unconventional T cells in relation to the frequency of corticosteroid exposure. However, we did not observe any associations between the duration of the treatment and the proportions of the analysed cells (Supplementary figure 1a and b). Caesarean section or pregnancy‐related complications such as maternal preeclampsia or chorioamnionitis did not influence the frequencies of the investigated cell types (data not shown).

### NK cell and γδ T‐cell characteristics are altered in extremely premature neonates

**Figure 4:**
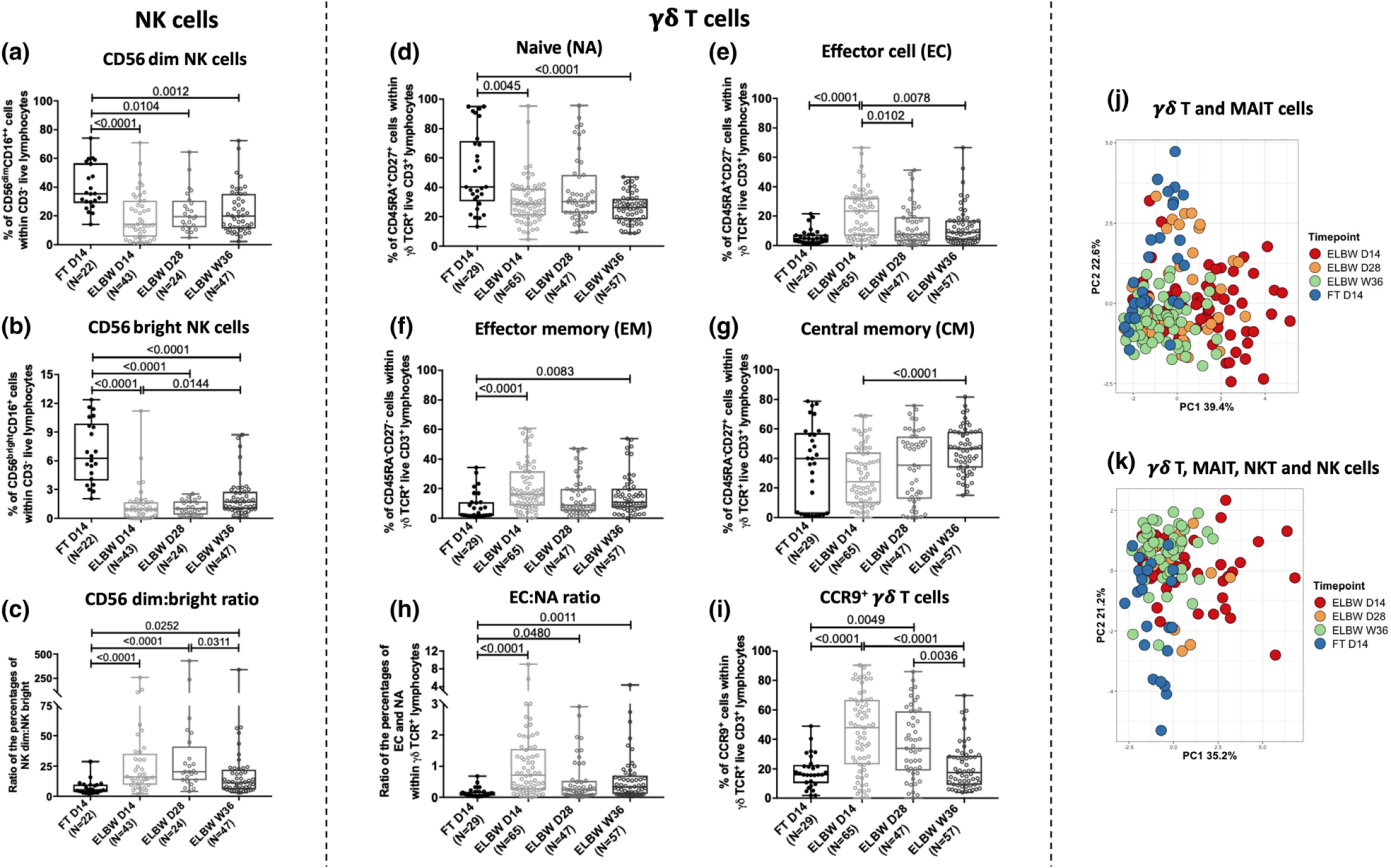
Extreme prematurity alters NK cell and γδ T‐cell phenotypical characteristics. The relative frequencies of **(a)** CD56^dim^‐ and **(b)** CD56^bright^ NK cells and **(c)** ratio between dim and bright NK cells at 14 days of age in ELGAN/ELBW and FT infants. **(d–g)** show the relative proportions of naïve (NA), effector (EC), effector memory (EM) and central memory (CM) γδ T cells in FT neonates and ELGAN/ELBW at the different sampling time points. The ratio of EC:NA γδ T cells is shown in **(h)**, and the frequencies of CCR9^+^ γδ T cells at 14 days of age are shown in **(i)**. The Mann–Whitney *U‐*test and Kruskal–Wallis test with Dunn's multiple comparison were used for group comparisons. Box and whisker plots show median as the central line and error bars represent minimum to maximum values for group comparison. PCA comparing the overall phenotypic characterisation in a longitudinal manner of MAIT and γδ T cells **(j)**, or the maturation over time of frequencies of all four investigated cell types **(k)**. FT neonates (blue), ELGAN/ELBW D14 (red), D28 (orange) and W36 (green).

NK cell subpopulations were defined according to their surface expression of CD56 and CD16 as CD56^bright^ (CD56^bright^CD16^+^) and CD56^dim^ (CD56^dim^CD16^++^) NK cells (Figure 2). The lower frequency of total NK cells observed in the ELGAN/ELBW group (Figure 4e) was reflected in both the CD56^dim^ and the CD56^bright^ subsets (Figure 4a and b; Supplementary table 1). The difference was most pronounced for the CD56 bright cells, which also resulted in a marked overall increase in the CD56^dim^:CD56^bright^ ratio (Figure 4c). Longitudinal analyses revealed that neither the subsets, nor the dim:bright ratio, attained similar frequencies as full‐term at day 28 and week 36 (Figure 4a–c; Supplementary table 1). We further characterised the naïve (NA), effector (EC), central memory (CM) and effector memory (EM) populations of γδ T cells, based on the surface expression of CD27 and CD45RA. Compared with FT neonates, NA γδ T cells in the ELGAN/ELBW group were significantly lower at day 14 of age, but they also remained low through the entire study period until week 36 (Figure 4d–h; Supplementary table 1). For terminally differentiated EC and EM cells, the pattern was the opposite, and these γδ T‐cell subsets were significantly higher in 14‐day‐old ELGAN/ELBW than in the FT infants. In line with the reduced frequencies of the NA subpopulation, the EC and EM γδ T cells remained high at all investigated time points (Figure 4e and f; Supplementary table 1). The proportion of CM γδ T cells increased with age, but did not differ between ELGAN/ELBW and FT neonates at week 36 (Figure 4g; Supplementary table 1). The shift in the γδ T‐cell subpopulations also resulted in a markedly higher EC:NA γδ T‐cell ratio in the ELGAN/ELBW at all time points investigated (Figure 4h). We further observed that the frequency of γδ T cells expressing CCR9 – a molecule associated with gut homing – was significantly higher in ELGAN/ELBW neonates at early time points (days 14 and 28), but approached frequencies similar to those of FT controls at week 36 (Figure 4i). Extended analysis by compiling the frequencies and characteristics of the investigated cell types confirmed that the unconventional T and NK cells in extremely premature infants differed markedly from FT neonates. Although some differences persisted throughout the study period, there was a clear trend of maturation with age, and at week 36, they were more similar to FT neonates (Figure 4j and k).

### The NK cell population at 14 days of life is influenced by culture‐proven sepsis onset before this time point

**Figure 5:**
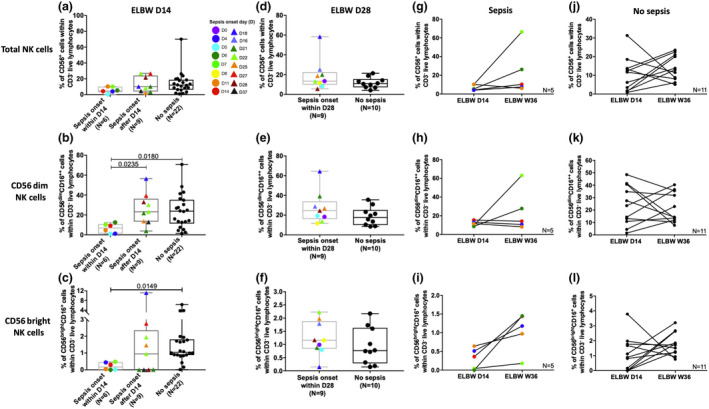
Sepsis accentuates the effect of extreme prematurity on the NK cell compartment. **(a–c)** show the proportions of total NK, CD56^dim^‐cell and CD56^bright^ NK‐cell frequencies, respectively, in 14‐day‐old ELGAN/ELBW with sepsis onset within 14 days of life, after day 14 of age, or no sepsis. **(d–f)** show the proportions of total NK, CD56^dim^‐cell and CD56^bright^ NK cell frequencies, respectively, in 28‐day‐old ELGAN/ELBW with sepsis onset within 28 days after birth or no sepsis. The proportions of total NK, CD56^dim^ and CD56^bright^ NK cells in paired samples from day 14 and week 36 are shown for cases with **(g–i)** or without **(j–l)** sepsis. Box and whisker plots show median as the central line and error bars represent minimum to maximum. The Mann–Whitney *U‐*test was used for group comparisons, and the Wilcoxon signed‐rank test was used to assess differences between pairs.

For the analysis of 14‐day samples in relation to culture‐proven sepsis, the preterm neonates were grouped as follows: culture‐proven sepsis with onset within 14 days of life, culture‐proven sepsis with onset after day 14 and no sepsis. No suspected sepsis with negative cultivation was included in the no sepsis controls in the following analyses. ELGAN/ELBW having sepsis onset within 14 days of life tended to have reduced percentages of total CD56^+^ NK cells in peripheral blood at 14 days of age, while the group with sepsis onset after day 14 did not differ from the no sepsis group (Figure 5a). The same observation was made for both CD56^dim^ and CD56^bright^ cell populations, where the group with sepsis onset within 14 days of life expressed significantly lower percentages of the respective NK cell population than the group with no sepsis (Figure 5b and c). This finding was unique for the early time point of 14 days, as the NK cell populations at day 28 were similar between sepsis and no sepsis cases (Figure 5d–f). Furthermore, paired analysis of neonates with sepsis onset within 14 days of life showed that the NK cell frequencies generally increased at week 36, particularly for the CD56^bright^ population (Figure 5g–i). For the ELGAN/ELBW preterm group with no sepsis, the pattern between the two time points was less clear (Figure 5j–l).

In our study, infants with positive blood culture were entailed in the inclusion criteria for sepsis, since it is considered to be the gold standard for sepsis diagnosis. Occasionally, no pathogens were detected in the blood even though the clinical signs and symptoms of sepsis were observed. Such suspected sepsis cases, negative for blood culture, were analysed separately and compared with the other groups (Supplementary figure 2a–f). Although the group of suspected sepsis was most similar to the group with culture‐positive sepsis with onset before day 14, it was not significantly different from the group without sepsis.

### Culture‐proven sepsis with an onset within 14 days of life associates with higher γδ T‐cell frequencies displaying an activated phenotype

**Figure 6:**
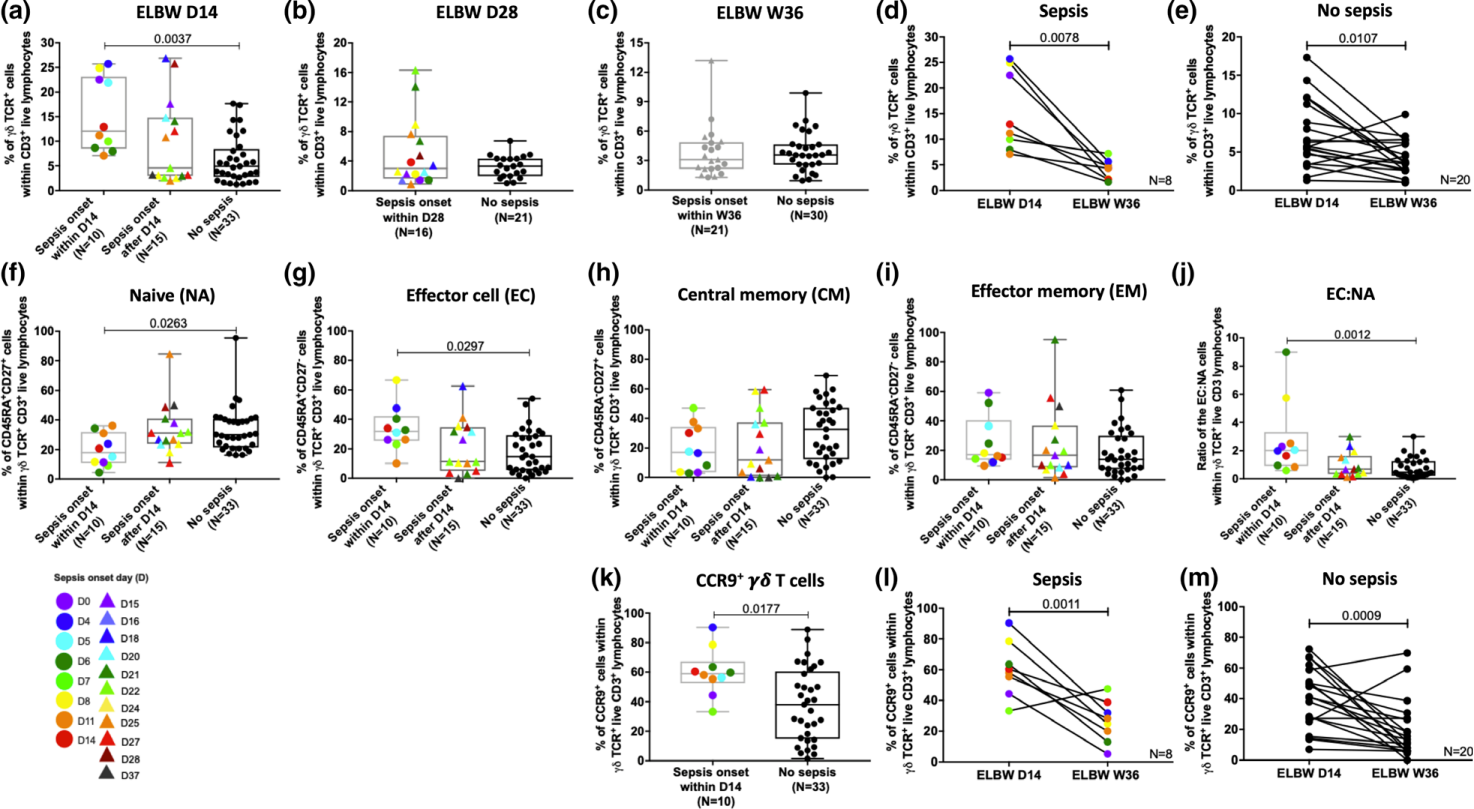
Increased γδ T‐cell frequencies, a rise in effector populations and an altered homing capacity are parameters associated with sepsis onset within 14 days of life. **(a–c)** The frequencies of γδ T cells from ELGAN/ELBW at day 14, day 28 and week 36, respectively, in relation to time of disease onset as indicated in the figure. The proportions of γδ T cells in paired samples from day 14 and week 36 are shown for cases with **(d)** or without **(e)** sepsis. **(f–i)** show the proportions of γδ T‐cell naïve, effector and memory subpopulations at 14‐day‐old ELGAN/ELBW, **(j)** shows the ratio between EC:NA γδ T cells and **(k)** displays the frequencies of CCR9^+^ γδ T cells in relation to sepsis onset. The proportions of CCR9^+^ γδ T cells in paired samples from day 14 and week 36 are shown for cases with **(l)** or without **(m)** sepsis. Box and whisker plots show median as the central line and error bars represent minimum to maximum. The Mann–Whitney *U‐*test and Kruskal–Wallis test with Dunn's multiple comparison were used for group comparisons. The Wilcoxon signed‐rank test was used to assess differences between paired samples.

There was a significantly higher percentage of γδ T cells in the 14‐day‐old ELGAN/ELBW having sepsis onset within 14 days of life in contrast to those with no sepsis within the first 14 days of life (Figure 6a). However, also this sepsis‐related finding seemed to be unique for the early time point, as the frequencies of γδ T cells did not differ between the sepsis and the no sepsis groups at day 28 and week 36 (Figure 6b and c). Paired analysis revealed that the frequencies of γδ T cells decreased significantly for extremely premature neonates between 14 days of life and week 36, regardless of sepsis diagnosis (Figure 6d and e). In the group with sepsis onset within 14 days of life, the γδ T‐cell effector cell frequencies were elevated, whereas the naïve γδ T‐cell proportions were reduced, leading to a significantly higher EC:NA ratio in this group (Figure 6f–j). Further, the proportion of γδ T cells expressing CCR9 was higher in the group of preterm neonates with a sepsis onset within the first 14 days of life (Figure 6k). Moreover, paired analysis showed that the frequency of CCR9‐expressing γδ T cells clearly declined from day 14 to week 36 in the preterm infants regardless of sepsis (Figure 6l and m). When including the suspected sepsis cases in the analyses, total γδ T‐cell frequencies were more similar to culture‐positive sepsis cases with onset within the first 14 days of life. However, there were no statistically significant differences between the group of suspected sepsis and any of the other groups (Supplementary figure 3a–i).

Sepsis, suspected sepsis or time of sepsis onset did not influence peripheral MAIT‐cell or NKT cell frequencies (Supplementary figure 4a and b). The role of NEC could not be evaluated for any of the cell types investigated, as only a maximum of four children with cells available for analysis at the D14 time point developed NEC within 14 days of life and these infants were excluded from the analyses in relation to sepsis.

### *Lactobacillus reuteri* supplementation has no impact on the composition of NK cells or non‐conventional T cells in ELGAN/ELBW neonates

**Figure 7:**
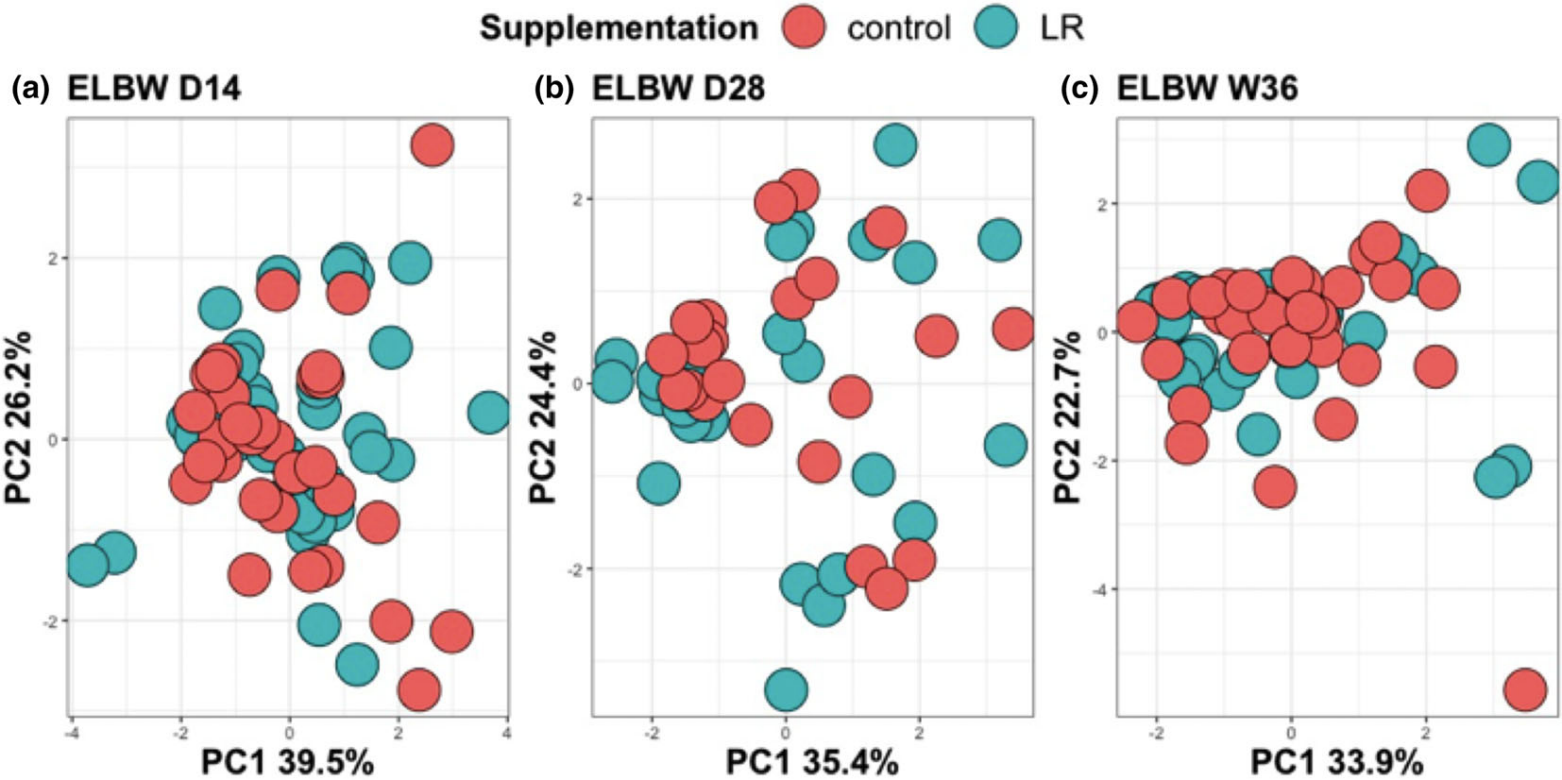
*Lactobacillus reuteri* supplementation has no effect on unconventional T cells. **(a–c)** show PCA plots at day 14, day 28 and week 36 of γδ T‐ and MAIT‐cell frequencies and phenotypic characteristics from ELGAN/ELBW receiving *L*. *reuteri* supplementation (green) or placebo (red).

Finally, we wanted to investigate whether the *L*. *reuteri* supplementation, administered to half of the preterm neonates included in this study, influenced any of the cells investigated above and could explain the substantial variation we sometimes observed within the groups. Notably, *L*. *reuteri* supplementation did not alter the relative proportions of MAIT cells, γδ T cells or their subpopulations at any of the time points investigated (Figure 7). Similarly, *L*. *reuteri* supplementation did not impact the circulating NK cell or NKT‐cell frequencies at day 14 and day 28, or week 36 (Supplementary table 2).

## Discussion

While several studies have investigated characteristics and functional properties of the preterm newborn immune system using cord blood, comprehensive and longitudinal studies on peripheral immunity in well‐characterised cohorts of extremely premature neonates are scarce. To the best of our knowledge, we are the first to report how the composition and functional phenotypes of peripheral unconventional T‐cell and NK cell populations change during the first months of life in a large group of extremely premature neonates from 14 days of life until they reach an age corresponding to PMW 36 + 0 (referred to as week 36). We have also related our findings to gestational age at birth, postnatal corticosteroid treatment and the occurrence of neonatal sepsis and pregnancy complications such as preeclampsia and chorioamnionitis. Finally, we have explored whether probiotic supplementation had an impact on the investigated cell types. Our findings highlight that in particular γδ T and NK cells are markedly affected by extremely preterm birth, alterations which are further manifested by sepsis onset within the first 14 days of life. Importantly, some of the investigated phenotypic characteristics relating to immune function persist during the postnatal period, at least until postmenstrual week 36.

The ontogeny of the immune system correlates with the developmental age of the foetus, and a competent immune system is suggested to develop during the second trimester.^19^, ^20^, ^21^ Due to the proactive care management at the border of viability in Swedish neonatal care,^22^ we had a unique opportunity to monitor the developmental trajectories of different circulating innate lymphocyte subsets in a cohort of extremely preterm neonates born as early as week 23. It is well established that prematurity in general has an impact on the immune compartment.^23^, ^24^, ^25^, ^26^ Still, study designs with a wide definition of prematurity, relatively small study groups and the frequent use of cord blood and lack of longitudinal approaches hamper the conclusions that can be drawn in terms of the very early stages of postnatal immune maturation. We recently reported on how conventional T‐cell subpopulations are significantly impacted by extreme prematurity, gestational age at birth, chorioamnionitis and necrotising enterocolitis.^25^ In this follow‐up study, we could demonstrate that extreme prematurity also had a profound effect on the frequencies but also on functional phenotypes of the peripheral innate lymphocyte compartment, with increased percentages of γδ T and NKT cells and markedly reduced NK cell frequencies at 14 days of age. In contrast, MAIT‐cell frequencies at 14 days of age were not altered by extreme prematurity, although their frequency in peripheral blood was generally very low, in line with what has been reported previously for cord blood MAIT cells from both term and preterm neonates.^27^, ^28^

The notable differences between FT and extremely premature neonates that we observe at 14 days of life could reflect the vulnerability of the lymphocyte compartment in early life (low frequencies of total CD3^+^ T cells and NK cells). The relatively high proportions of γδ T and NKT cells are likely to reflect a developmental aspect, as the conventional T‐cell compartment is lagging behind this early during development. Accordingly, the frequencies of unconventional T cells constitute a larger proportion of the CD3^+^ T‐cell compartment in premature infants. In our study subjects, the total CD3^+^ T‐cell compartment was significantly influenced by gestational age at birth, probably reflecting a lower thymic output of αβ T cells at this early age, while there was no correlation between frequencies of any of the other investigated cell populations and gestational age at birth. However, we cannot exclude that the environment is also contributing the findings we observe, as we have not investigated the cells before day 14 in the extremely premature infants. It could reflect the exposures associated with their stay at the NICU during the first 2 weeks of life, such as ventilatory support, recurrent deoxygenation and hypotension and increased exposure to infections (high frequencies of γδ T and NKT cells).

The low proportions of conventional T cells in these extremely premature infants clearly make them more dependent on their unconventional T‐cell populations and NK cells. The finding of significantly lower NK cell frequencies in the preterm infants is interesting and in line with previous studies with both full‐term and preterm neonates.^29^, ^30^, ^31^ It should be noted that we have not performed functional analysis of NK cells in this study, so we cannot rule out that functions such as cytokine production, and cytotoxic abilities are further affected and dependent on gestational age at birth in the ELGAN/ELBW neonates. Although gestational age at birth did not impact NK cell frequencies at day 14 among the preterm neonates, NK cell frequencies remained low during the entire study period. Together with a hampered conventional T‐cell compartment, this could contribute to the increased vulnerability and enhanced susceptibility to infection also after the first critical months of life for these infants, which has been reported in observational studies.^32^, ^33^ Circulating innate lymphocytes are markedly affected by sepsis in adult patients,^34^ with accelerated apoptosis of NK cells leading to a decreased number of NK cells with a hypo‐responsive status that persists for several weeks.^34^, ^35^, ^36^, ^37^ The impact of sepsis in human neonates is much less studied, in particular in extremely premature infants. Notably, we here observed that the neonates developing sepsis within the first 14‐day period of life had lower frequencies of NK cells at 14 days of age than those that developed sepsis later during the study period, or did not develop sepsis at all. We also report that the low frequencies of NK cells were true for both CD56^dim^ and CD56^bright^ subpopulations, also in agreement with findings in adult sepsis patients.^38^ We further noted that the dim:bright NK cell ratio was higher, which could be indicative of a skewing towards a cytotoxic profile of the NK cells in preterm neonates with sepsis, although the possible functional consequences of this were not further investigated here. Interestingly, the low NK cell frequencies were not seen at the later time points (day 28 and week 36), again highlighting the sensitivity of the first 2 weeks of life for the extremely preterm neonates.

In contrast to the NK cells, γδ T‐ and NKT‐cell frequencies were significantly higher in the extremely premature neonates at 14 days of life. γδ T cells have been described as key players in fighting pathogens, with the subgroup of Vδ2‐expressing γδ T cells being involved in the response to both parasites and bacteria.^39^, ^40^ It was recently demonstrated that the γδ T‐cell population, and the Vδ2 subpopulation in particular, was strongly influenced by postnatal life in premature neonates and also influenced by the microbial environment.^41^ In previous work, we reported that although extremely premature and FT neonates had similar proportions of Vδ2‐positive γδ T cells at 14 days of age, their functional characteristics were impaired in the preterm group.^42^ However, in the study presented here, we did not further study the contribution of Vδ chain expression, but rather different degrees of maturity based on naïve, effector and memory subpopulations. A characterisation of the γδ T cells in the ELGAN/ELBW group revealed an activated phenotype with an enhanced effector:naïve cell ratio together with strongly upregulated expression of CCR9. The CD45RA^+^CD27^‐^ effector γδ T cells are described as terminally differentiated with an enhanced ability for cytotoxicity and cytokine production,^43^ features that are important for good pathogen responses. As γδ T cells are known to be important in maintaining gut integrity and homeostasis,^44^ it is possible that the large population of CCR9‐positive γδ T cells detected in the circulation at day 14 in the preterm group are destined for the gut, a process that might not be completed at this early phase of pregnancy. Our observation on the reduction in circulating CCR9^+^ γδ T‐cell frequencies with age could further support that this is a maturational process. This would have been interesting to further evaluate in relation to development of NEC, but this was not possible because of a lack of power.

The role of γδ T cells in human sepsis is not fully elucidated, with an imbalance and functional alterations of the γδ T‐cell population in adult sepsis reported in some studies,^45^, ^46^ but not in others.^35^ In the extremely premature neonates, there was an even higher proportion of γδ T cells in the neonates after they had developed sepsis, and the group with sepsis onset within the first 14 days of life had the highest EC:NA ratio, which agrees with what has been reported for adult sepsis patients^45^ and could suggest that these cells took an active role during the infection. We did not observe any associations between sepsis and MAIT‐ or NKT‐cell frequencies in our cohort, in contrast to what has been reported for adult sepsis patients.^34^ However, as we did not perform functional studies of these cells, it is therefore not possible to completely rule out any sepsis‐associated effects on these cell types.

We should acknowledge the fact that in all our analyses, we did not perform in‐depth analyses of the cell types investigated here. It is possible that detailed analyses, for example Vδ chain usage in γδ T cells or the use of MR1 and CD1d tetramers to identify MAIT and NKT cells, respectively, would have generated further insights regarding the roles of these cell types at this early period of life and also in relation to clinical parameters. Considering our data which highlight the relative importance of unconventional T cells and NK cells during this vulnerable period of life, this warrants further detailed investigations of these cells and how they contribute and/or are affected by infections in premature infants.

One of the major differences between ELGAN/ELBW and FT neonates with possible consequences for the immune system in the early postnatal period is the use of corticosteroids. Differential effects on adult human circulating lymphocytes by intravenous betamethasone have been reported.^47^, ^48^ Further, chorioamnionitis and maternal preeclampsia are reported to influence the immune characteristics of the offspring.^25^, ^49^, ^50^ In our study, we did not see any correlation between innate lymphocyte frequencies and their characteristics and the use or duration of postnatal intravenous corticosteroid treatment, chorioamnionitis or preeclampsia, suggesting that our results are not just reflecting corticosteroid effects and/or pregnancy‐related complications.

The role of probiotic supplementation for immune function and complications such as sepsis has been studied in preterm neonates. A pooled meta‐analysis that compared ‘probiotics’ with ‘placebo’ or ‘no probiotics’ showed that probiotic supplementation resulted in a statistically significant reduction in the incidence of late onset of sepsis.^51^ However, several studies fail to show a clear association with immune function and sepsis risk.^52^, ^53^ Still, these studies are often difficult to compare due to major differences in inclusion criteria, choice of probiotic strain, read‐outs etc. In our cohort, half of the participating premature neonates had received daily supplementation with *L*. *reuteri* from birth and during the entire study period. Notably, *L*. *reuteri* supplementation had no impact on any of the parameters studied here at any of the three time points investigated.

In conclusion, this is the first longitudinal study of peripheral blood innate lymphocytes in a well‐defined group of extremely premature neonates, where we clearly demonstrate that characteristics of both γδ T and NK cells are strongly influenced by extreme prematurity and that these alterations are further enhanced by sepsis onset during the first 2 weeks of life. The data presented here suggest that a compromised NK cell compartment may contribute to the increased risk for immune‐mediated severe morbidity such as necrotising enterocolitis, retinopathy of the prematurity and poor neurological outcome as well as infections both during and beyond the neonatal period in these infants.

## Methods

### Study design and participants

The present study was part of the prospective, double‐blinded, randomised controlled, multi‐centre trial PROPEL (Prophylactic Probiotics to Extremely Low Birth Weight Premature Infants), evaluating the effect of probiotic *Lactobacillus (L.)* *reuteri* DSM 17938 on feeding tolerance, growth, severe morbidities and mortality (ClinicalTrials.gov ID NCT01603368). The study design has been described in detail elsewhere^54^ and was approved by the Ethics Committee for Human Research in Linköping, Sweden (Dnr 2012/28‐31, Dnr 2012/433‐32). Infants born between gestational week 23 + 0 and 27 + 6 and with a birthweight less than 1000 g were eligible for enrolment within 3 days after delivery. The infants were characterised using comprehensive clinical data including perinatal data, growth, feeding intolerance, treatment, antibiotics and mild and severe morbidities collected daily in a study‐specific case report form until a time point corresponding to postmenstrual week (PMW) 36 + 0 (Table 1).

**Table 1:** Background and clinical characteristics of the study participants.

ELGAN/ELBW (*n* = 97)	Sepsis within 14 days of life (*n* = 14)	No sepsis within 14 days of life (*n* = 83)	*P* ^a^
Gestational age, weeks, mean (SD)	24.9 (1.3)	25.4 (1.2)	0.168
Birthweight, g, mean (SD)	709 (124)	723 (140)	0.717
Birthweight *z*‐score, mean (SD)	−0.73 (0.90)	−1.16 (1.32)	0.241
Small for gestational age (weight < 2 SD), *n* (%)	1 (7%)	22 (27%)	0.176
Male, *n* (%)	7 (50%)	46 (55%)	0.776
Apgar at 5 min, mean (SD)	5.5 (2.2)	6.5 (2.5)	0.171
Infants from multiple pregnancy, *n* (%)	8 (57%)	28 (34%)	0.093
Antenatal corticosteroids, *n* (%)	13 (93%)	82 (99%)	0.269
Caesarean section, *n* (%)	7 (50%)	54 (65%)	0.280
Maternal smoking, *n* (%)	0 (0%)	7 (8%)	0.588
Preeclampsia, *n* (%)	1 (7%)	9 (11%)	1.00
Chorioamnionitis, *n* (%)	5 (36%)	15 (18%)	0.156
Preterm premature rupture of membranes, *n* (%)	5 (36%)	25 (30%)	0.756
Maternal antibiotics, *n* (%)	10 (71%)	47 (56%)	0.385
Inclusion site, Stockholm/Linköping, *n*/*n* (%/%)	13/1 (93%/7%)	50/33 (60%/40%)	0.017
Antibiotics during first week, *n* (%)	14 (100%)	83 (99%)	1.00
Antibiotics during second week, *n* (%)	14 (100%)	69 (83%)	0.209
Days on antibiotics, mean (SD)	36.4 (13.9)	27.2 (14.4)	0.028
Betamethasone treatment, *n* (%)	2 (14%)	27 (33%)	0.217
Betamethasone treatment before day 14, *n* (%)	1 (7%)	5 (6%)	1.00
Betamethasone treatment before day 28, *n* (%)	2 (14%)	19 (23%)	0.727
Days on mechanical ventilation, mean (SD)	21.7 (18.7)	20.3 (17.1)	0.785
NEC, Bells stage II–III, *n*/*N* ^b^ (%)	4/14 (29%)	8/83 (10%)	0.068
Bronchopulmonary dysplasia, *n*/*N* ^b^ (%)	7/13 (54%)	52/80 (65%)	0.537
Intraventricular haemorrhage, grade 3–4, *n*/*N* ^b^ (%)	3/14 (21%)	8/82 (10%)	0.199
Retinopathy of prematurity, grade 3–5, *n*/*N* ^b^ (%)	5/13 (38%)	10/81 (12%)	0.031
Death before 360 days, *n* (%)	1 (7%)	3 (4%)	0.469

^a^ The Student's *t*‐test was used to compare means and the chi‐square test was used to compare frequencies.

^b^ *N* is the number of infants that survived until diagnosis could be set.

A sepsis diagnosis required positive blood and/or cerebrospinal fluid culture, clinical deterioration and laboratory‐confirmed inflammatory response. Detailed clinical signs and symptoms of sepsis for both culture‐positive (pos) and culture‐negative (neg) cases were presented in Supplementary table 3. The bacteria detected in positive culture included Gram‐negative rods such as *Escherichia coli* and *Klebsiella* and Gram‐positive *Streptococcus* group B, *Enterococcus*, *Staphylococcus aureus* and coagulase‐negative *Staphylococci*. Sepsis onset within 14 days of life was more common in the children included in Stockholm, and these children more frequently developed retinopathy and they also received longer antibiotic treatment. There was also a tendency for a higher occurrence of NEC in the group with sepsis onset within 14 days of life (Table 1).

Blood sampling from the preterm infants at D14 and D28 and at PMW 36 + 0 as well as from 29 full‐term (FT) infants (born between PMW 38 and 42) at 14 days of age was performed as depicted in Figure 1. In all subsequent figures, the following abbreviations are used: FT D14 (samples from full‐term infants at day 14 after birth), ELBW D14, ELBW D28 and ELBW W36 (samples from ELGAN/ELBW infants at day 14, day 28 and PMW 36 + 0, respectively). Whenever it was possible based on cell availability, we performed longitudinal analysis with paired samples in order to see possible changes over time in the same individuals. According to cell availability, the comparison was made either between three (D14, D28 and W36) or two time points (D14 and W36). When evaluating cellular characteristics at 14 days of life in relation to sepsis, we grouped the 14‐day‐old premature infants as having sepsis onset from 0 to 14 days after birth (denoted as sepsis onset *within* D14 in the figures) and sepsis onset after 14 days of birth (denoted as sepsis onset *after* D14 in the figures). For analysis of cells collected at 28 days, comparisons were made between infants with sepsis onset within day 28 of life and infants having no sepsis. There were too few cases with sepsis onset after day 28 of life to include them as a separate group. Infants with suspected sepsis but that were culture‐negative were excluded from the analysis.

### Blood collection and isolation of peripheral blood mononuclear cells

Peripheral blood was drawn by venous (or arterial) puncture. At D14 and D28, 0.8 mL blood was collected in custom‐made 1.5‐mL Eppendorf tubes with heparin as anticoagulant. The heparin was bought from the local hospital pharmacy. At W36, 2.5 mL blood was collected in 4‐mL Vacuette tubes (Hettich, Sweden). Peripheral blood mononuclear cells (PBMCs) were isolated by Ficoll‐Hypaque (GE Healthcare Bio‐sciences AB, Uppsala, Sweden) gradient separation from the FT infants (*N* = 29 at D14) and from all ELGAN/ELBW infants (*N* = 97) from whom a sufficient blood volume was obtained (*N* = 72 at D14, *N* = 52 at D28 and *N* = 59 at W36). Briefly, the blood was diluted in prewarmed RPMI‐1640 (GE Healthcare Life Sciences, Hyclone Laboratories, UT). The diluted blood was layered on the top of the Ficoll and centrifuged for 30 min. The white cloudy interface containing the PBMCs was collected and washed three times in warm RPMI‐1640 and resuspended in freezing medium containing 40% RPMI‐1640, 50% foetal calf serum (Sigma‐Aldrich, St. Louis, MO) and 10% DMSO (Sigma‐Aldrich). Cells were gradually frozen in a freezing container (Mr. Frosty™; Thermo Scientific) and kept in liquid nitrogen until further analysis.

### Flow cytometry and antibodies

Frozen PBMCs were thawed gently, washed three times and seeded into the 96‐well tissue culture plate (Costar, Corning Incorporated, ME) at a concentration of 0.25 × 10^6^ cells/well. The cells were rested for 2 h at 37°C with 5% CO_2_ before staining. Cells were then transferred to V‐shaped staining plates and stained with the LIVE/DEAD Fixable Dead Cell Stain Kit‐Aqua (Life Technologies, Eugene, OR) according to the manufacturer's instructions. Blocking of cell surface Fc receptor was done with 10% human serum. Two different FACS panels were used to characterise NK cells and unconventional T cells. Staining of cell surface markers was performed using the following antibodies from BioLegend (San Diego, CA): CD3 (clone: OKT3), CD161 (clone: HP‐3G10), CD27 (clone: O323), TCR Vα7.2 (clone: 3C10), TCR γδ (clone: B1), CD56 (clone. MEM‐188), CD16 (clone: 3G8), TCR Vα24‐Jα18 (clone: 6B11), CD45RA (clone: HI100), CD4 (clone: OKT4) and CCR9 (clone: L053E8). Stained cells were washed, resuspended in FACS wash buffer and acquired using FACS Verse instrument and FACS Suite software (BD Biosciences). Data analysis was performed using FlowJo software (Ashland, OR). The antibody panels and the gating strategies for FACS analysis are shown in Figures 1, 2, respectively.

### Statistical analysis

GraphPad Prism 7 (GraphPad Software, La Jolla, CA) was used for the statistical analyses. Graphs were displayed either as box and whiskers showing min to max, or as symbol and lines. The results were shown as medians with interquartile ranges (IQR) for continuous variables with skewed distributions shown in the figures and in the supplementary tables. The Mann–Whitney *U‐*test and the Wilcoxon signed‐rank test were used to assess differences between groups and pairs, respectively. The non‐parametric Kruskal–Wallis test followed by Dunn's multiple comparisons test was performed to compare age‐related differences within the ELGAN/ELBW group of preterm infants. The Spearman correlation test was used to analyse correlation between variables shown in the figures. Pearson's chi‐square test was used for categorical outcome variables. Fisher's exact test was used when the observed frequency for any cell was less than five. Results were considered as significant when *P* < 0.05, and actual *P*‐values are displayed in each figure. The principal component analyses (PCA) were performed in SPSS V25. Different combinations of cell subsets frequencies were reduced to two principal components. The amount of variance in the data explained by one component is mentioned as a percentage on the axis. The total variance in the data that is explained by the two components combined is mentioned in the graphs. The validity of the PCA was checked with the Kaiser–Meyer–Olkin measure of sampling adequacy and the Bartlett's test of sphericity.

## Conflict of interest

Thomas Abrahamsson has received honoraria for lectures and a grant for the present trial from Biogaia AB. Maria C Jenmalm has also received honoraria for lectures from Biogaia AB. Eva Sverremark‐Ekström has received honoraria for lectures and a grant for another research project from BioGaia AB. The other authors have no conflict of interest to declare.

## Author contributions

**Khaleda Rahman Qazi:** Data curation; Formal analysis; Investigation; Methodology; Writing‐original draft. **Georg B Jensen:** Data curation; Investigation; Writing‐review & editing. **Marieke van der** **Heiden:** Formal analysis; Writing‐review & editing. **Sophia** **Björkander:** Methodology; Writing‐review & editing. **Giovanna** **Marchini:** Investigation; Writing‐review & editing. **Maria C** **Jenmalm:** Conceptualization; Writing‐review & editing. **Thomas** **Abrahamsson:** Conceptualization; Funding acquisition; Investigation; Resources; Writing‐review & editing. **Eva** **Sverremark‐Ekström:** Conceptualization; Funding acquisition; Project administration; Resources; Supervision; Writing‐original draft; Writing‐review & editing.
