# Supplementary material for: Extreme prematurity and sepsis strongly influence frequencies and functional characteristics of circulating γδ T and natural killer cells
Source: Clin Transl Immunology. 2021 Jun 10;10(6):e1294. doi: 10.1002/cti2.1294 (PMC8192243; doi:10.1002/cti2.1294)
Supplement: Supplementary file 1 [file CTI2-10-e1294-s001.pdf]

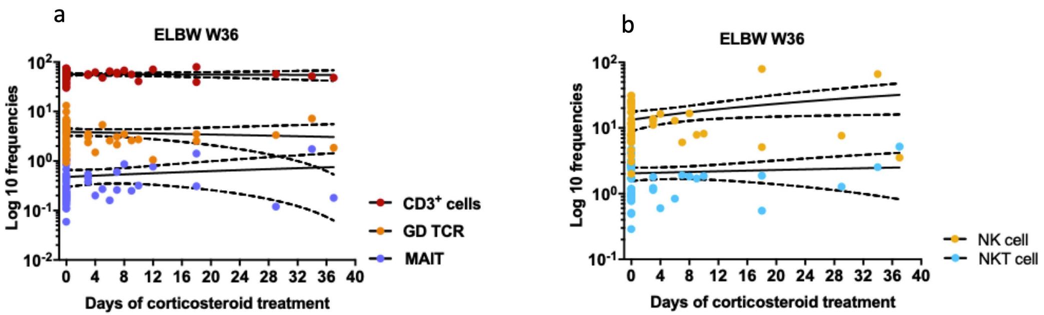

Supplementary figure 1. Long term corticosteroid exposure has no effect on the frequencies of the NKT- or NK cell compartments at week 36. Correlations between the days of corticosteroid exposure and the percentages of **(a)** $CD3^+$ T-, γδ T-, MAIT cells and **(b)** NK- and NKT cells in ELGAN/ELBE at week 36.

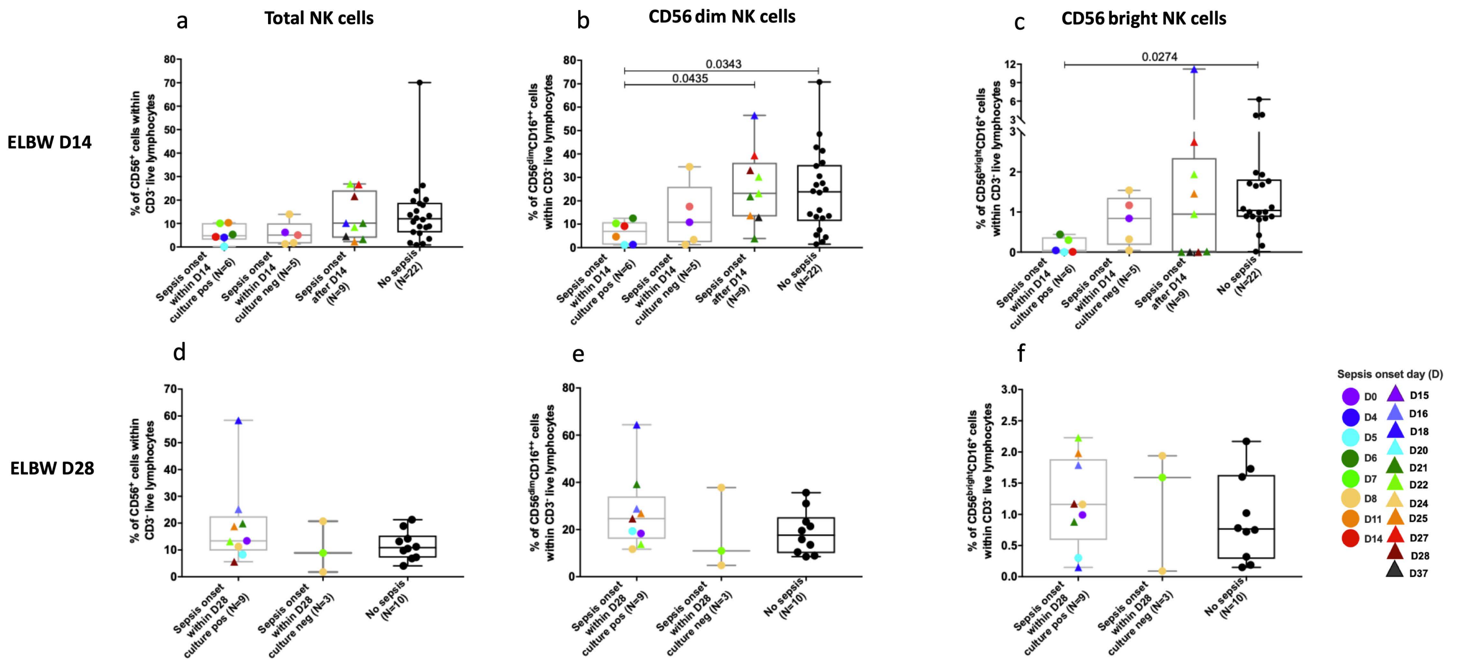

Supplementary figure 2. The frequencies of NK cells and their subpopulations in culture negative (culture neg) sepsis cases do not differ from any of the other groups**. (a-c)** show the proportions of total NK-, CD56dim – and CD56bright NK-cell frequencies respectively, in 14-day old ELGAN/ELBW with sepsis onset within 14 days of life, culture pos and culture neg, after day 14 of age, or no sepsis. **(d-f)** show the proportions of total NK-, CD56dim – and CD56bright NK-cell frequencies respectively, in 28-day old ELGAN/ELBW with sepsis onset within 28 days after birth (culture pos and neg) or no sepsis.

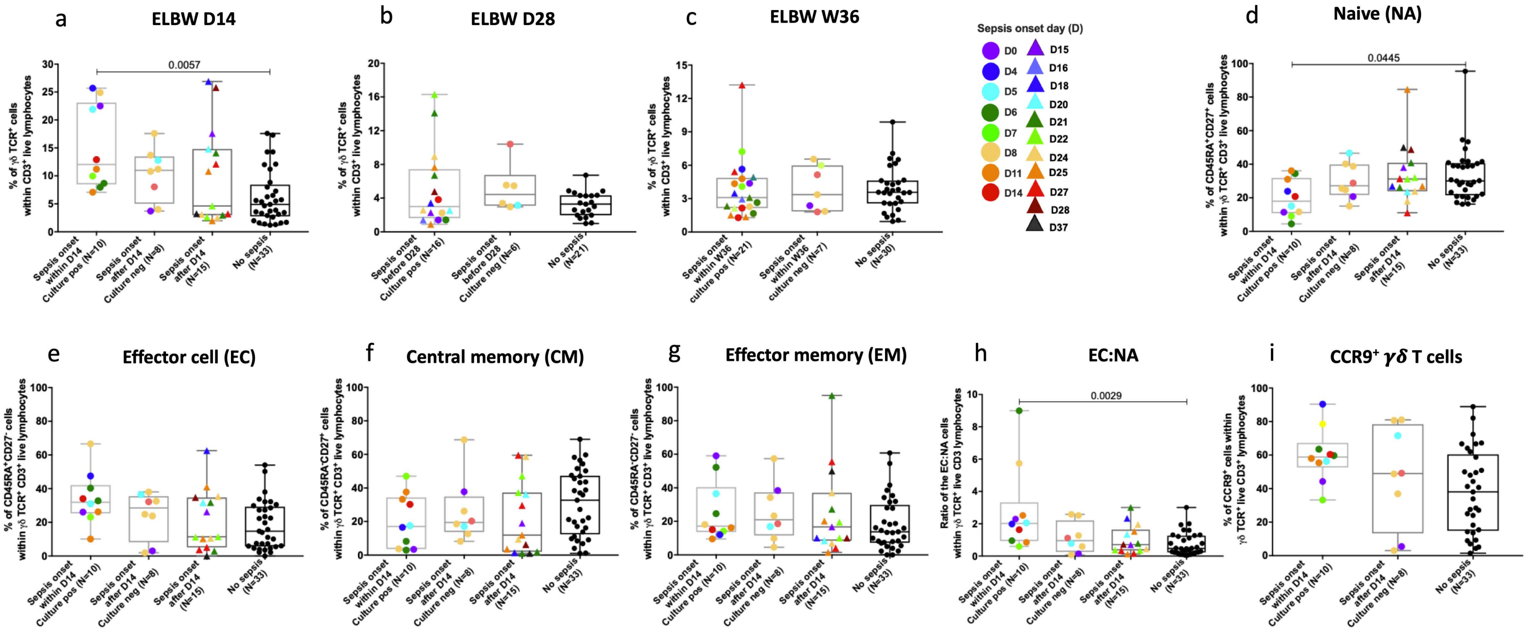

Supplementary figure 3. The frequencies of $\gamma\delta$ T cells and their subpopulations do not differ between culture negative (culture neg) sepsis cases and any of the other groups. **(a-c)** show the proportions of total $\gamma\delta$ T cell in 14-day, 28-day and 36 PMW old ELGAN/ELBW children in relation to time of disease onset as indicated in the figure. **(d-g)** show the proportions of $\gamma\delta$ T cell naïve, effector and memory subpopulations in the PBMCs of 14-day old ELGAN/ELBW, **(h)** shows the ratio between EC:NA $\gamma\delta$ T cells and **(i)** displays the frequencies of CCR9+ $\gamma\delta$ T cells in relation to sepsis onset (both culture pos and neg).

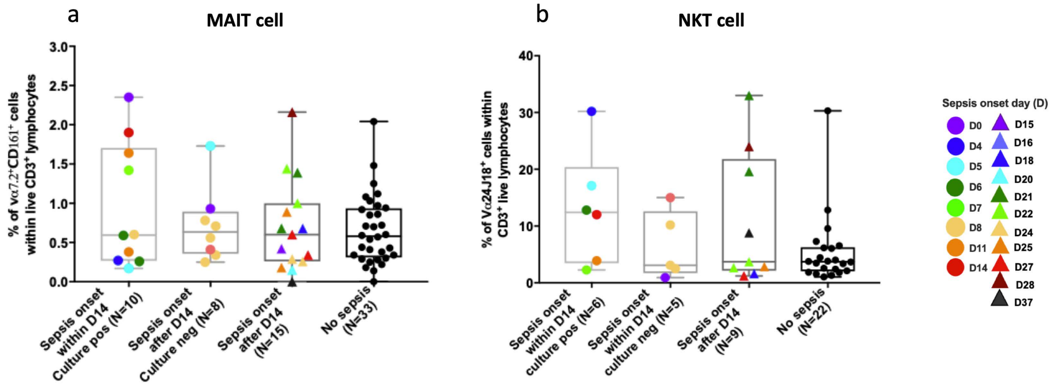

Supplementary figure 4. Peripheral MAIT- and NKT cells are not affected by sepsis. Comparison of the proportions of **(a)** MAIT- and **(b)** NKT cells from 14-day old ELGAN/ELBW neonates in relation to the time of sepsis onset including culture negative sepsis cases.

Supplementary table 1. Proportions of NK and unconventional T cells[1]

Cell populations	Percentages of	FT D14	ELBW D14	ELBW D28	ELBW W36
%CD3+ T[a]	Median	76.2	20.4****	56.5****	57.3****
	IQR	7.2	48.27	23.55	18.7
%$\gamma\delta$ T cells[b]	Median	1.91	6.38****	3.15	3.5
	IQR	1.67	9.1	2.72	2.55
%$\gamma\delta$ NA[c]	Median	40.3**	28.55	30.2	26****
	IQR	41.05	18.25	25.7	14.25
%$\gamma\delta$ EC[c]	Median	4.62	23.25****	7.56	8.87
	IQR	5.2	25.41	16.33	13.28
%$\gamma\delta$ EM[c]	Median	2.28	16.2	8.84	10.8**
	IQR	9.05	23.99	14.92	12.76
%$\gamma\delta$ CM[c]	Median	40	20.4	35.4	46.7
	IQR	55.97	35	42.2	24.15
%MAIT cells[b]	Median	0.68	0.59	0.43	0.26
	IQR	1.47	0.64	0.45	0.34
%NKT cells[b]	Median	1.58	3.81****	1.48	1.76
	IQR	1.53	7.78	2.59	1.77
%Total NK cells[d]	Median	33.8	9.4****	11.25****	10.7****
	IQR	18.38	12.03	11.35	10.01
%CD56 Dim NK cells[d]	Median	35.35	16****	19.4**	19.8***
	IQR	27.7	22.96	18.3	23.9
%CD56 Bright NK cells[d]	Median	6.28	0.89****	1****	1.71****
	IQR	5.93	1.62	1.38	1.83

[1] Values are given as percentage of median and interquartile range (IQR). Significant differences with full-term (FT) controls are shown as asterisks. *$P \leq 0.05$. **$P \leq 0.01$. ***$P \leq 0.001$. ****$P \leq 0.0001$. [a]Percentages of cells within live lymphocytes. [b]percentages of cells within live $CD3^+$ cells. [c]percentages of cells within total $\gamma\delta$ T cells. [d]Percentages of cells within $CD3^-$ cells.

Supplementary table 2. Percentages of NKT and NK cell subsets in the PBMCs of ELBW preterm neonates supplemented with *Lactobacillus reuteri* or placebo[1]

Cell populatins		**ELBW D14**		**ELBW D28**		**ELBW W36**		*P*-Value
		L. reuteri (N=21)	Placebo (N=22)	*L. reuteri* (N=10)	Placebo (N=14)	*L. reuteri* (N=20)	Placebo (N=28)	
NKT cells	Median	4.49	3.73	1.66	1.32	2.52	1.69	NS
	IQR	9.31	7.69	3.88	1.93	1.92	1.57	
Total NK cells	Median	8.48	10.25	10.85	12.25	11.8	9.25	NS
	IQR	11.96	12.18	11.08	11.98	18.34	10.47	
CD56 Dim NK cells	Median	23.2	14	23	18.8	19.45	16.75	NS
	IQR	25.29	21.18	19.83	19.5	19.13	25.39	
CD56 Bright NK cells	Median	0.95	0.86	1.59	0.76	1.61	1.72	NS
	IQR	1.93	0.97	1.03	1.05	2.17	1.60	

[1] Values are given as percentage of median and interquartile range (IQR).

Supplementary table 3. Characteristics of the sepsis cases.

Individual	Day of sepsis onset	Study group	Inclusion site	GW	Sex	Delivery mode	PPROM	Chorioamnionitis
*Culture-positive sepsis**								
1	22	*L. reuteri*	Linköping	27	Boy	Caesarean	No	No
2	4	Placebo	Stockholm	24	Girl	Vaginal	No	No
3	33	Placebo	Linköping	26	Girl	Caesarean	No	No
4	21	*L. reuteri*	Stockholm	24	Boy	Caesarean	Yes	No
5	22	*L. reuteri*	Stockholm	27	Girl	Caesarean	Yes	No
6	14	Placebo	Stockholm	23	Boy	Vaginal	No	No
7	22	*L. reuteri*	Linköping	23	Girl	Vaginal	Yes	No
8	5	*L. reuteri*	Stockholm	23	Girl	Vaginal	No	No
9	27	*L. reuteri*	Linköping	23	Boy	Caesarean	No	No
10	24	Placebo	Linköping	25	Boy	Caesarean	No	No
11	7	*L. reuteri*	Stockholm	24	Boy	Vaginal	No	Yes
12	15	*L. reuteri*	Stockholm	26	Boy	Caesarean	No	No
13	0	*L. reuteri*	Stockholm	26	Girl	Caesarean	Yes	Yes
14	8	Placebo	Stockholm	25	Boy	Caesarean	No	No
15	28	*L. reuteri*	Stockholm	23	Girl	Vaginal	No	Yes
16	24	Placebo	Stockholm	27	Boy	Caesarean	No	No
17	6	*L. reuteri*	Stockholm	25	Boy	Vaginal	Yes	No
18	25	*L. reuteri*	Linköping	25	Boy	Caesarean	Yes	No
19	18	Placebo	Linköping	26	Girl	Caesarean	No	No
20	20	*L. reuteri*	Stockholm	26	Boy	Caesarean	Yes	Yes
21	6	*L. reuteri*	Stockholm	27	Girl	Caesarean	Yes	No
22	16	Placebo	Stockholm	26	Boy	Caesarean	No	No
23	11	*L. reuteri*	Stockholm	24	Boy	Vaginal	No	No
24	25	*L. reuteri*	Stockholm	24	Girl	Caesarean	No	No
25	25	*L. reuteri*	Linköping	26	Boy	Vaginal	No	No
26	37	*L. reuteri*	Stockholm	25	Boy	Caesarean	Yes	Yes
27	41	*L. reuteri*	Stockholm	23	Boy	Vaginal	No	Yes
28	21	Placebo	Stockholm	23	Boy	Vaginal	No	No
29	27	*L. reuteri*	Linköping	25	Girl	Caesarean	No	No
30	0	Placebo	Stockholm	24	Girl	Caesarean	Yes	Yes
31	6	*L. reuteri*	Stockholm	26	Girl	Caesarean	No	Yes
32	7	*L. reuteri*	Linköping	23	Girl	Caesarean	No	No
33	11	Placebo	Stockholm	24	Boy	Vaginal	No	Yes
34	20	Placebo	Stockholm	26	Boy	Vaginal	Yes	No
35	9	Placebo	Stockholm	25	Boy	Caesarean	Yes	No
*Culture-negative sepsis***								
1	7	*L. reuteri*	Stockholm	27	Boy	Caesarean	No	No
2	8	Placebo	Linköping	23	Boy	Vaginal	No	No
3	67	Placebo	Stockholm	23	Boy	Vaginal	No	No
4	8	*L. reuteri*	Linköping	24	Boy	Caesarean	No	No
5	0	Placebo	Linköping	23	Girl	Vaginal	Yes	Yes
6	7	*L. reuteri*	Stockholm	25	Girl	Caesarean	Yes	Yes
7	52	Placebo	Linköping	24	Boy	Vaginal	Yes	No
8	49	*L. reuteri*	Linköping	23	Girl	Vaginal	No	No
9	8	*L. reuteri*	Linköping	24	Boy	Caesarean	No	No
10	24	*L. reuteri*	Stockholm	26	Girl	Caesarean	No	No
11	13	Placebo	Stockholm	25	Boy	Vaginal	No	No
12	13	*L. reuteri*	Stockholm	25	Girl	Caesarean	No	No
13	8	Placebo	Stockholm	25	Boy	Vaginal	No	No
14	8	Placebo	Linköping	23	Girl	Vaginal	No	No
15	5	Placebo	Stockholm	24	Girl	Caesarean	No	No
16	37	*L. reuteri*	Stockholm	25	Boy	Caesarean	Yes	Yes
17	22	*L. reuteri*	Linköping	25	Girl	Vaginal	No	No
18	45	*L. reuteri*	Stockholm	24	Boy	Caesarean	No	No

*For culture-positive sepsis a diagnosis required a positive blood and/or cerebral spinal fluid culture together with a minimum of 2 out of 4 of the following: 1. White blood cell (WBC) count $\leqq 5$ or $\geqq 20 \times 10^9$ cells/L; 2. Total platelet count $\leqq 100 \times 10^9$ cells/L; 3. CRP $\geqq 15$ mg/L; 4. Newly recognised apnea, elevated oxygen demand or requirement of respiratory support. In case of a positive culture of coagulase-negative staphylococci, the culture-positive sepsis diagnosis required two separate positive blood cultures with the same antibiotic resistance pattern and/or a central venous/arterial line prior to sepsis onset together with at least 1 of the laboratory criteria (1-3) mentioned before and clinical deterioration (criteria 4). **For culture-negative sepsis a diagnosis required a negative blood culture and at least 3 out of the mentioned criteria (1-4) for culture-positive sepsis. GW, gestational week at birth; PPROM, preterm premature rupture of membranes; *L. reuteri*, *Lactobacillus reuteri.*
